# Temporal Variation and Assembly Process of Fish Communities in a Typical Canalized River After the 10-Year Fishing Ban

**DOI:** 10.3390/biology14091208

**Published:** 2025-09-07

**Authors:** Qiang Qin, Yuanfeng Li, Peng Chen, Ting Wei, Jianghaoyue Xu, Fubin Zhang, Tong Zhou

**Affiliations:** 1 College of Environmental Science and Engineering, China West Normal University, Nanchong 637009, China; L17721985130@163.com (Y.L.); 19160926860@163.com (P.C.); weiting_t1@163.com (T.W.); xujianghaoyue@163.com (J.X.); sczhangfubin@163.com (F.Z.); 2 Yangtze River Fisheries Research Institute, Chinese Academy of Fishery Sciences, Wuhan 430223, China; zhoutong@yfi.ac.cn

**Keywords:** fish resource, community assembly, fisheries management, biodiversity conservation, the Jialing River

## Abstract

The Jialing River is a representative and typical canalized tributary in the upper Yangtze River basin. In the context of the 10-year fishing ban, it is imperative to elucidate the dynamics and assembly of fish communities in the Jialing River. In this study, field surveys were conducted during the flood and dry seasons from 2021 to 2025 at the Cangxi section of the Jialing River. Fifty fish species belonging to three orders and six families were collected in the study area, with *Chanodichthys oxycephaloides*, *Hemibarbus labeo*, *Xenocypris davidi*, and *Siniperca chuatsi* identified as dominant species. In addition, fish communities in this region exhibited significant temporal variations, clustering into flood season and dry season groups (*p* < 0.05). The result suggested that diversity indices were consistently higher in the flood season than the dry season and an increasing trend was observed during the sampling periods. Community assembly analysis revealed that the fish communities were mainly characterized by phylogenetic clustered structure, indicating environmental filtering as the deterministic process in the study area. Despite the steady recovery of fish biodiversity and resource following the fishing ban in the study area, further attention must be directed to other anthropogenic disturbances, especially habitat fragmentation. This study provides a scientific reference for fisheries management after the fishing ban in the Jialing River.

## Simple Summary

As the largest tributary of the upper Yangtze River basin, the Jialing River plays an important role in the protection of fish biodiversity and resource. In recent years, anthropogenic disturbances (e.g., river canalization) have posed severe crises for fish species in the Jialing River. However, studies on the fish community in the Jialing River after the implementation of the fishing ban were scarce. In this study, we explored the dynamics and assembly of fish communities in a representative section of the Jialing River after the 10-year fishing ban. The study indicated that fish biodiversity and resources have steadily recovered after the fishing ban, while some endemic and endangered fish species recovered relatively slowly in the study area. It also reminded us that attentions should be paid to the impacts of anthropogenic disturbance and corresponding measures should be taken to promote the restoration of fish resource. Moreover, comprehensive and integrated analyses should be conducted in further studies.

## 1. Introduction

The river ecosystem is an important component of aquatic ecosystems, which contribute significantly to biodiversity and resource conservation [1,2]. However, human activities in recent decades have posed serious threats to river ecosystems and have created severe crises for aquatic organisms [3,4]. Petts [5] and Saunders et al. [6] identified river ecosystems as historically crucial ecosystem service providers, which are particularly vulnerable to anthropogenic disturbances. Moreover, river ecosystems also provide indispensable habitats for fish. For instance, the Yangtze River basin is one of the most specific river ecosystems with complex habitats in China, harboring 424 species of freshwater fishes and 194 species of endemic fishes only distributed in the Yangtze River [7,8,9]. Unfortunately, the Yangtze River basin has faced intensive exploitation and overfishing, leading to a drastic decline in fish resource and a fishless crisis of fish biodiversity [10]. As the river ecosystems of the basin are under enormous disturbance pressure, some fish species such as *Hucho bleekeri* and *Onychostoma angustistomata* are at risk of extinction [11]. In order to protect fish resources, the Chinese government imposed a 10-year fishing ban in the Yangtze River basin (http://www.cjyzbgs.moa.gov.cn/tzgg/201912/t20191227_6334009.htm, accessed on 27 December 2019). In this context, Xiong et al. [12] and Li et al. [13] performed several studies on fish communities in the mainstream, but the fish community responses to this fishing ban in the tributaries of the Yangtze River basin are still poorly understood.

The Jialing River has a total length of 1120 km and a catchment area of 160,000 km^2^ [14]. In the past, the Jialing River was a major fishing region in southwest China due to its abundant fish resource. Liu [15] and Jiang et al. [16] assessed fishery resource prior to the complete fishing ban and found that the annual catch of fish in the Jialing River exceeded 5000 tons. The Jialing River is also a key area for fish biodiversity conservation in the Yangtze River basin. Zeng et al. [17] and Qing [18] reported that there were 156 fish species distributed in the Jialing River basin, and 56 species were endemic to the Yangtze River. Specifically, the Jialing River became a typical canalized tributary in the upper Yangtze River basin owing to high-density dam development. Canalization and overfishing are considered the main drivers causing severe challenges to native fishes in the Jialing River. As early as the 1980s, Deng et al. [19] recommended that corresponding measures should be taken to protect fish resources in the river. The Cangxi section is located at the boundary between the upper and middle Jialing River, maintaining relatively abundant fish resource and exhibiting distinctive characteristics in biodiversity [18]. Meanwhile, this section also lies between the Cangxi and Shaxi dams, serving as a microcosm of the canalization of the Jialing River. Although previous studies investigated fish community structure in the Jialing River, little attention has been paid so far to fish community dynamics and assembly after the 10-year fishing ban [16,20,21].

Fish community dynamics and assembly are fundamental to ecological research and have consistently intrigued ecologists. Among these, fish community dynamics respond to habitat changes and anthropogenic disturbances, directly reflecting the condition of river ecosystems [22,23]. The temporal variation in fish communities underpin community dynamics, which have been extensively described in case studies. For example, Gonzalez et al. [24] found that fish community structure in the Orinoco River changed with flood dynamics over time. Gao [25] concluded that the fish community composition changed from rushing water species to lentic water species following the impoundment of the Three Gorges Dam. Liu et al. [26] also reported that the fish community in the Chishui River had distinct temporal changes, and fish resource has recovered rapidly under dam-free and fishing-ban conditions. Fish community assembly also reveals mechanisms of coexistence and biodiversity maintenance, which are of great significance for the conservation and restoration of river ecosystems. The focus of community assembly continuously revolved around niche-based theory and neutral theory [27,28,29,30]. Niche-based theory explains community assembly through limiting similarity and environmental filtering, while neutral theory attributes community assembly to stochastic drifting [31,32,33]. And the advances in molecular biology have also enabled an accurate description of pedigree relationships and the prediction of community assembly. Thus, the mechanisms of community assembly have been widely explored through empirical and theoretical studies [34,35]. In the context of river canalization and the fishing ban, comprehensive observation of fish community dynamics and assembly in the Jialing River is required.

In this study, we conducted a continuous fish community survey in a typical canalized section of the Jialing River after the implementation of the 10-year fishing ban. Based on the sampling data, the main objectives were to (1) document the species composition of fish communities in the study area; (2) analyze the temporal variation in fish communities after the implementation of the fishing ban in the Jialing River; (3) examine the assembly process of fish communities in the canalized section of the Jialing River. From the study on representative section of the Jialing River, we want to explore questions against the backdrop of river canalization: what impacts does the fishing ban have on the composition and assembly of fish communities, and what effects does the fishing ban have on the restoration of fish biodiversity and resource? Our findings will provide valuable insights for the conservation of fish biodiversity following the fishing ban in the study area, and will offer scientific references for the restoration of fish resource in other sections of the canalized river.

## 2. Materials and Methods

### 2.1. Study Area

The Jialing River originates from the northern Qinling Mountain and flows through four Chinese provinces: Shaanxi, Gansu, Sichuan and Chongqing [36]. Generally, the whole river can be divided into three sections, the upstream with deep valleys ranges from Fengxian to Guangyuan; the midstream holds circular channels and extends from Guangyuan to Hechuan; and the downstream has gentle riverbeds and stretches from Hechuan to Chaotianmen of Chongqing [14]. A total of 16 cascade dams have been constructed in the mainstream of the Jialing River, and the Chinese government has imposed a 10-year fishing ban on this river from 1 January 2021 to 31 December 2030 (http://www.cjyzbgs.moa.gov.cn/tzgg/201912/t20191227_6334009.htm, accessed on 27 December 2019).

**Figure 1:**
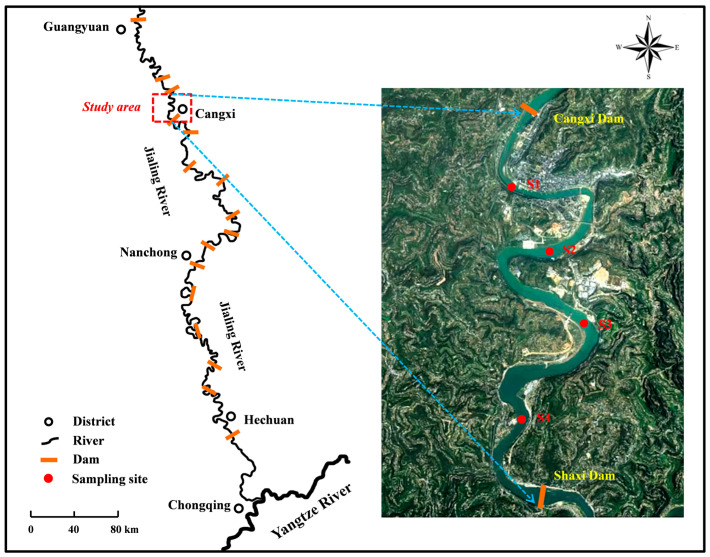
The map of the study area in the Jialing River.

In this study, samples were collected from the Cangxi section in the midstream of the Jialing River (31°37′–31°45′ N, 105°55′–105°56′ E), covering a river section approximately 12 km in length (Figure 1). In total, we set up 4 sampling sites (S1–S4) in this section. The study area representing the canalized section of this river is located between the Cangxi and Shaxi Dams, serving as a microcosm of the canalization of the Jialing River. The water quality of this river section has been consistently maintained at Grade II of China’s surface water quality standard. The study area is also characterized by hilly-mountainous topography and a subtropical monsoon climate, where the water supply mainly comes from the precipitation concentrated during the flood seasons [37].

### 2.2. Sample Collection

The field surveys were conducted biannually in the flood season (June–July) and the dry season (November–December) from 2021 to 2025. The fish samples were collected using gillnets with mesh sizes of 4 cm, 6 cm, 8 cm, and 10 cm (100 m long × 2 m high × 8 pcs and 200 m long × 2 m high × 2 pcs) and trap nets with a mesh size of 0.5 cm (30 m long × 4 pcs) in each sampling site. In addition, gillnets and trap nets were placed horizontally within the scope of approximately 2000 m in each sampling site and put in the water for average 4 h at night during each sampling time. Once captured, all samples were identified to the species level following Ding [38] and Chen [39]. Then, the total length (*TL*) to the nearest 0.1 cm and body weight (*BW*) to the nearest 0.1 g for fish samples were recorded in the field. Finally, the fish samples were preserved in a 10% formalin solution and stored at the College of Life Science, China West Normal University.

### 2.3. Phylogeny Construction

In this study, we employed the DNA barcoding gene of cytochrome *c* oxidase I (COI) to construct a phylogeny quantifying the phylogenetic relationships of fishes from the study area. The available sequence data of COI for 50 fish species were downloaded from the GenBank database (https://www.ncbi.nlm.nih.gov/, accessed on 29 June 2025) (Supplementary Table S1). In addition, all procedures including sequence extraction, alignment, and concatenation were performed in Phylosuite, which integrated multiple software programs for phylogenetic analyses. After that, maximum likelihood (ML) methods inferred by IQ-TREE with 1000 standard bootstrap replicates were applied to phylogeny construction. Finally, the phylogenetic distance matrix of fish species was obtained on the basis of the phylogeny by TreeSuite (Supplementary Figure S1). All analyses above were performed in Phylosuite v1.2.1 [40].

### 2.4. Statistical Analysis

The index of relative importance (*IRI*) was used to evaluate the fish species dominance and was calculated using $IRI=\left(N_{i}\%+W_{i}\%\right)\times F_{i}\%\times 10^{4}$, where *N_i_*% is the quantity percentage of species *i*, *W_i_*% is the weight percentage of species *i*, and *F_i_*% is the occurrence frequency of species *i*. *IRI* ≥ 1000 are dominant species, 1000 > *IRI* ≥ 100 are subdominant species, 100 > *IRI* ≥ 10 are companion species, and *IRI* < 10 are rare species. In addition, the International Union for Conservation of Nature (IUCN) category mainly referred to China’s Red list of Biodiversity: Vertebrates [41]. The trophic guild identification was mainly based on the feeding characteristics assigned from the literature [15,42,43].

Margalef’s richness index (*d*), Shannon’s diversity index (*H*), Pielou’s evenness index (*J*), and Simpson’s dominance index (*D*) were employed to analyze the diversity variations of fish communities [44,45,46,47]. Margalef’s richness index (*d*) was calculated using $d=\frac{\left(S\;-1\right)}{lnN}$; Shannon-Wiener’s diversity index (*H*) was measured using $H=-\sum p_{i}lnp_{i}$; Pielou’s evenness index (*J*) was computed using $J=\frac{H}{lnS}$; and Simpson’s dominance index (*D*) was estimated using $D=1-\sum {p_{i}}^{2}$, where *S* is the total number of fish species, *N* is the total quantity of fishes, and *p_i_* is the proportion of species *i* to the total quantity of fishes. One-way ANOVA was employed to detect significant differences of diversity indices between the groups of sampling periods.

Hierarchical cluster analysis based on Bray–Curtis similarity was performed to identify fish community dynamics during the sampling periods [48]. The community data were square root–transformed before cluster analysis to reduce the skewness caused by outliers. Then, non-metric multidimensional scaling (NMDS) was used to verify the degree of similarity according to the Bray–Curtis similarity matrix [49]. And analysis of similarity (ANOSIM) was also employed to examine whether there were significant differences between the groups of sampling periods [50]. Finally, the similarity of percentage analysis (SIMPER) was conducted to identify the contribution of each species to dissimilarity at different groups of sampling periods [7].

The assembly process of fish communities was inferred by the metrics of the net relatedness index (*NRI*), which represents the phylogenetic dispersion of coexisting species [51]. *NRI* is the standardized effect size (*SES*) of the mean pairwise phylogenetic distance (*MPD*) for species in each community [52]. It was calculated using $NRI=\frac{-1\times \left({MPD}_{obs}-{MPD}_{null}\right)}{sd\left({MPD}_{null}\right)}$, where *MPD_obs_* is the observed value of the mean pairwise phylogenetic distance among all pairs of species; *MPD_null_* represents the null distribution of the mean pairwise phylogenetic distance where species was shuffled 999 times across the tips of the phylogeny; and *sd* (*MPD_null_*) is the standard deviation of the null distribution for mean phylogenetic pairwise distance. The independent-swap null model was employed to generate 1000 random communities constituting a null distribution [53]. The standardized effect sizes and significance (two-tailed test) of the observed values could be calculated by comparing with the null distribution. A negative *NRI* indicates that species relatedness is lower than the expected null hypothesis and community assembly is overdispersed; a positive *NRI* shows that species relatedness is higher than the expected null hypothesis and community assembly is clustered; and a value of *NRI* close to the expected null hypothesis suggests that community assembly should be random. Student’s t-test was used to test significant differences of *NRI* between the flood and dry season groups.

Statistical analyses were performed in Primer 6.1.13 [50], SPSS 20.0 [54], OriginPro 9.4.2 [55], and package ‘picante’ in R 4.5.0 [56,57].

## 3. Results

### 3.1. Fish Species Composition

In this study, a total of 7,512 individuals with a total weight of 2,767,612.2 g were collected during the sampling periods from 2021 to 2025 in the study area. The fish samples belonged to three orders, six families, and fifty species (Table 1). Among these, Cyprinidae and Bagridae were dominant families which consisted of 35 species (70.0%) and 8 species (16.0%), and Sinipercidae had 3 species (6.0%), Siluridae had 2 species (4.0%), and Catostomidae and Gobiidae had 1 species (2.0%), respectively. Moreover, *C. oxycephaloides*, *H. labeo*, *X. davidi*, and *S. chuatsi* were dominant species. *Hypophthalmichthys nobilis* showed the largest total length (110 cm) and body weight (12,015 g), while the lowest total length (0.9 cm) and body weight (2.1 g) were found in *Rhinogobius giurinus* (Table 1).

According to feeding habits, the fish species could be divided into four trophic guilds. The guild of omnivores contained 19 species, the guild of herbivores held 12 species, the guild of invertivores included 10 species, and the guild of piscivores had 9 species (Table 1). The ICUN category of *Myxocyprinus asiaticus* was critically endangered (CR) species; *Onychostoma macrolepis*, *Xenocypris fangi*, *Procypris rabaudi*, and *Tachysurus pratti* were vulnerable (VU) species; *Onychostoma simum* and *Tachysurus dumerili* were near threatened (NT) species; *Tachysurus emarginatus* and *Bangana rendahli* were data deficient (DD) species, and the others were least concern (LC) species (Table 1). *M. asiaticus*, *P. rabaudi*, and *O. macrolepis* were listed as second-class national protected species of China, and *Hemiculter tchangi*, *X. fangi*, *Rhinogobio cylindricus*, *Saurogobio punctatus*, *P. rabaudi*, and *B. rendahli* were endemic fishes of the upper Yangtze River.

### 3.2. Temporal Variation in Fish Communities

**Figure 2:**
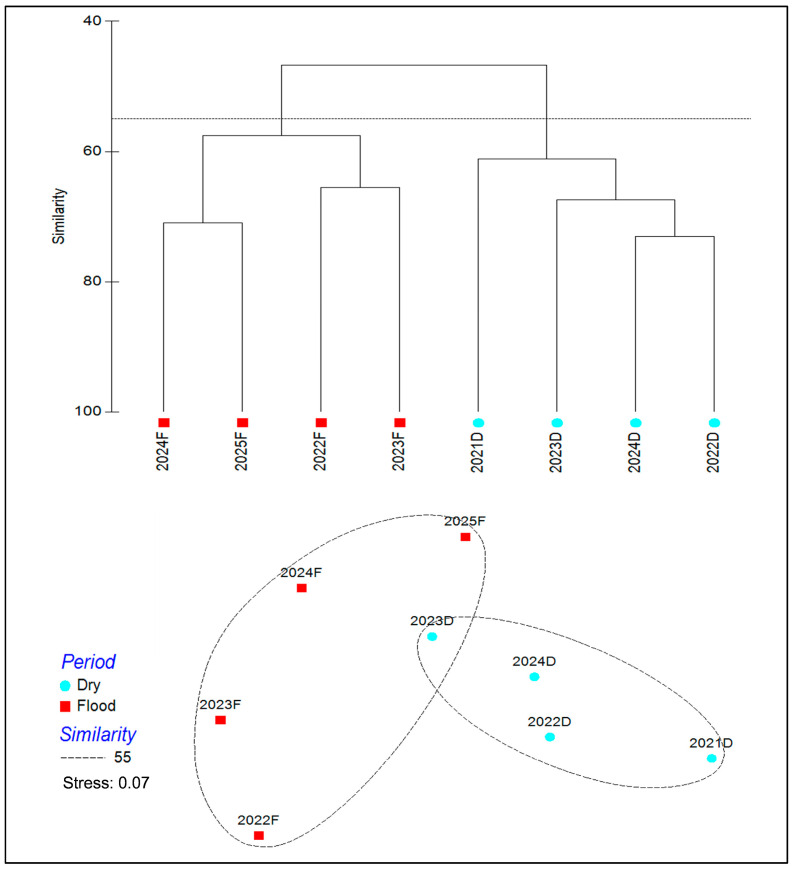
The hierarchical cluster analysis based on Bray–Curtis similarity for sampling periods with a 55% similarity level, and non-metric multidimensional scaling (NMDS) ordination for sampling periods with a stress of 0.07.

According to cluster analysis based on Bray–Curtis similarity, during the sampling period from 2021 to 2025, fish communities could be classified into two groups (with a 55% similarity level): the flood season group and the dry season group (Figure 2). NMDS ordination showed that the results supported dividing fish communities during the sampling periods into two categories under a stress of 0.07, which was in good agreement with the classification from cluster analysis (Figure 2). Moreover, the ANOSIM test demonstrated that there was a significant difference in fish species composition between the flood season group and dry season group (global *R* = 0.49; *p* < 0.05). SIMPER analysis showed that the average dissimilarity of the flood season group and the dry season group was 40.31%, and the result suggested that the fish species (e.g., *S. chuatsi*, *C. oxycephaloides*, *H. labeo*, *Pseudobrama simoni*) were mainly responsible for the differences observed.

**Figure 3:**
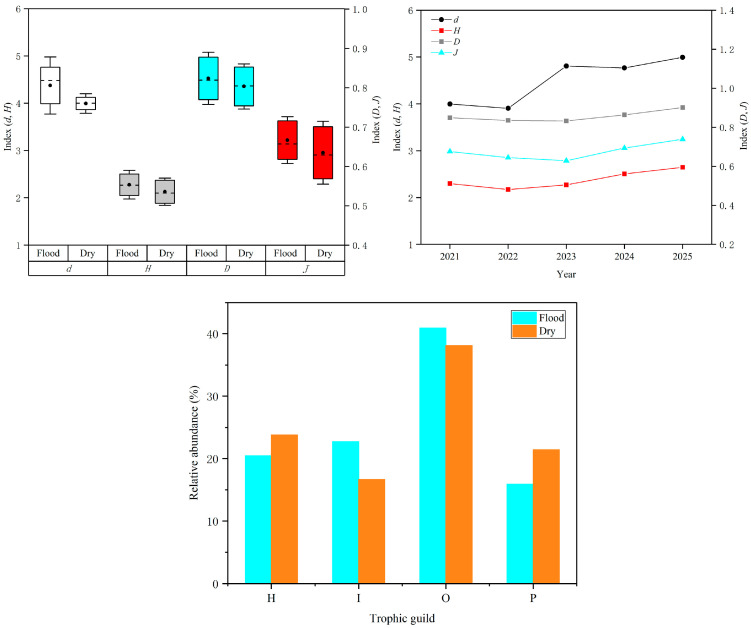
The diversity indices of fish communities in the flood and dry seasons and the trend of variation during the sampling periods; the relative abundance of fish species belonging to different trophic guilds in the flood and dry seasons in the study area.

For the flood and dry season groups, the mean ± SD of Margalef’s richness index (*d*) was 4.38 ± 0.60 in the flood season group and 3.99 ± 0.21 in the dry season group; the mean ± SD of Shannon–Wiener’s diversity index (*H*) was 2.28 ± 0.30 in the flood season group and 2.13 ± 0.29 in the dry season group; the mean ± SD of Pielou’s evenness index (*J*) was 0.67 ±0.06 in the flood season group and 0.64 ± 0.08 in the dry season group; and the mean ± SD of Simpson’s dominance index (*D*) was 0.82 ± 0.07 in the flood season group and 0.80 ± 0.06 in the dry season group (Figure 3). We can conclude that the community diversity indexes in the flood season were higher than those in the dry season, but no significant differences were detected for community diversity indices between the flood and dry season groups (*p* > 0.05), and the community diversity indices showed an upward trend from 2021 to 2025 (Figure 3). Additionally, fish species belonging to trophic guilds of omnivores and invertivores appeared more frequently in the flood season, while fish species belonging to trophic guilds of herbivores and piscivores were collected more frequently in the dry season (Figure 3).

### 3.3. Assembly Process of Fish Communities

**Figure 4:**
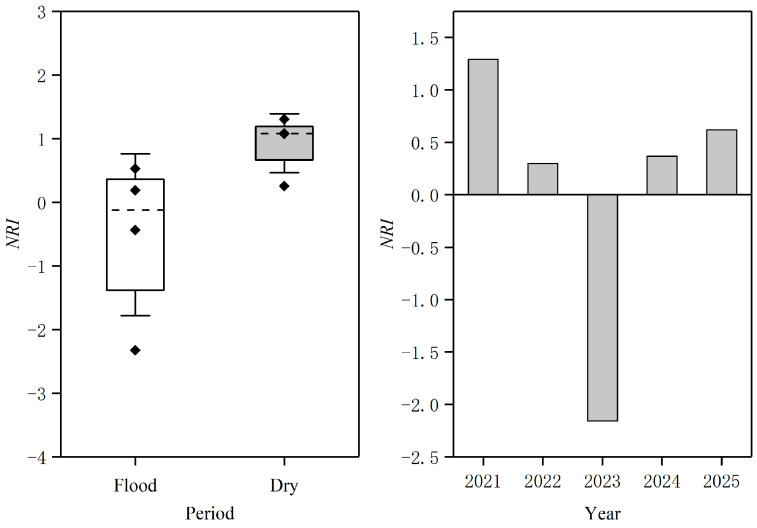
The assembly process of fish communities during the sampling periods and years in the study area.

In this study, the assembly process of fish communities in the study area was determined by phylogenetic dispersion tests. The results showed that the values of *NRI* for fish communities during the sampling periods varied from −2.32 to 1.30 (Figure 4). Among them, six sampling periods had positive values and two sampling periods had negative values, indicating that fish community assembly was mainly controlled by environmental filtering, while only two sampling periods were significant regarding the expected null hypothesis (*p* < 0.05) (Figure 4). Specifically, the mean values of *NRI* were −0.51 in the flood season group and 0.93 in the dry season group, which implied that phylogenetic overdispersion in the flood season group was more prominent, and fish community assembly was dictated by phylogenetic clustering in the dry season group. However, there was no significant difference for *NRI* between the flood and dry seasons (*p* > 0.05). We also found that the phylogenetic clustered structure also dominated the assembly process of fish communities from 2021 to 2025 (Figure 4). Overall, the assembly of fish communities in the study area was mainly characterized by phylogenetic clustering, indicating that environmental filtering plays a key role in this region.

## 4. Discussion

In this study, we conducted a continuous field survey in a representative section of the Jialing River after the implementation of the fishing ban. As a result, fifty fish species were collected in the study area during the sampling periods from 2021 to 2025. Zhou et al. [36] used environmental DNA technology to identify 99 fish species in the entire Jialing River. This suggests that the sampling region is relatively speciose and makes it an ideal area for studying fish community dynamics and assembly. This study also showed that *C. oxycephaloides*, *H. labeo*, *X. davidi*, and *S. chuatsi* were dominant species, and their populations grew relatively rapidly compared with the studies conducted by Qing [18] and Zhang [20] before the fishing ban. Thus, we inferred that river canalization in the Jialing River provided more favorable habitats for these dominant species, which are adapted to lentic and eurytopic hydrological conditions [58,59]. In addition, other factors such as climate change that affect the species composition of fish communities should be comprehensively considered in subsequent research. We also found a higher proportion of omnivorous fish in the composition of fish species, which is similar to canalized rivers in the upper Yangzte River. For example, Zou et al. [60] revealed that fish communities in the midstream of the Tuojiang River were dominated by omnivorous fish, accounting for 63.22% of the total species. Cheng et al. [61] also discovered that dam construction in the Wujiang River has dramatically altered the water conditions, resulting in the fish community being mainly characterized by lentic and omnivorous fish species. Moreover, the fish species collected in the study area included two national protected species, six endemic fish species of the upper Yangtze River, and five endangered or vulnerable species in the ICUN category. Liu et al. [26] indicated that the occurrence rates of key protected species increased, and 11 native species that had disappeared for years reappeared after the fishing ban in the Chishui River, demonstrating that eliminating anthropogenic pressures significantly contributes to the recovery of fish resource. We summarized the studies in the Jialing River and found that threatened and endemic fish species have begun to recover since the fishing ban, though their populations still remain low, primarily due to human activities such as dam construction [16,18,20,62].

Moreover, our study shows that fish communities in the study area exhibited significant temporal variation during the sampling periods from 2021 to 2025, clustering into distinct flood season and dry season groups. This indicates that the observed fish community variation was possibly driven by hydrological dynamics caused by the subtropical monsoon climate in the Jialing River basin [63,64]. This was consistent with most river ecosystems in the monsoon climate regions of East Asia [65,66]. Zhang et al. [67] reported significant seasonal changes in fish community structure between the dry and flood seasons in the Duliu River. Additionally, our study suggested that diversity indices were consistently higher in the flood season than the dry season and showed an increasing trend during the sampling periods. The seasonal variation in diversity indices observed in present study aligns with findings from the Juma River and the Yangzte River [68,69]. We also found that diversity indices in the present study had a certain increase compared to an earlier study before the fishing ban [18]. In combination with our finding of a gradual improvement in diversity indices, we conclude that fish resource and biodiversity continue to recover after the fishing ban in the Jialing River. Liu et al. [26] also confirmed that the ban effectively promoted the recovery of fish resource in the Chishui River. Several studies reminded us that the ban is an important measure for fish biodiversity protection and resource restoration, especially in river ecosystems with severe anthropogenic disturbances [70,71].

The rules of community assembly are primarily represented by the process of limiting similarity, environmental filtering or neutral effects [30]. In this study, the assembly process of fish communities was mainly characterized by phylogenetic clustering on the temporal scale, indicating environmental filtering as the dominant deterministic process in the study area. It also reminded us that fish communities were composed of closely related species with similar characteristics to adapt specific habitats under the effect of environment filtering [51,72]. Yang et al. [73] explored fish community assembly in the midstream of the Yangtze River and concluded that strong environmental filtering drove fish community assembly in the Three Gorges Reservoir. Yet, Qin [74] demonstrated that competitive exclusion dominates fish community assembly in the Chishui River, the only undammed tributary in the upper Yangtze basin. So we can infer that the effect of environmental filtering caused by dam construction serves as a primary process of fish community assembly in the canalized rivers. It hindered survival and reproduction of the lotic-adapted fish species and caused population decline, while fish species with specific traits that favor lentic water can coexist stably in the canalized river [36,75]. However, the correlated analysis for environmental variables of canalization was not covered in this study, and the corresponding data should be integrated to identify the key factors for the effect of environmental filtering. At present, studies on the assembly process of fish communities in canalized rivers remain scarce. Thus, this study enriches our understanding of the mechanisms of fish community assembly.

This study reveals that fish biodiversity and resource in the Jialing River have steadily recovered after the complete fishing ban. However, some endemic and endangered fish species (e.g., *M. asiaticus*, *P. rabaudi*, *B. rendahli*) were still identified infrequently during the sampling periods, indicating that their populations are recovering slowly. Numerous studies have demonstrated that overfishing was one of the major pressures affecting fish resource before the fishing ban, and other anthropogenic disturbances should not be ignored [76,77,78]. In the context of the fishing ban, the impact of anthropogenic disturbances on fish diversity and resource has not been completely eliminated [79,80]. The findings in our study also implied that anthropogenic disturbances such as dam construction may be responsible for variation in species composition and process of community assembly in the canalized river. Thus, we propose corresponding suggestions for the biodiversity conservation and resource recovery of fish in the study area: (1) Continuous attention should be paid to the population dynamics of endangered and endemic fish in future studies; (2) Comprehensive surveys and integrated analyses should be conducted to provide scientific references for the fisheries management; (3) Targeted regulation and restoration of habitats should be carried out to minimize the impact of existing anthropogenic disturbances.

## 5. Conclusions

In summary, our study revealed that the fish communities exhibited certain temporal variation and fish biodiversity has steadily recovered after the implementation of the fishing ban in the study area. However, some endemic and endangered fish species maintain small populations and are recovering relatively slowly. We determined that environmental filtering is the dominant deterministic process in the study area, reminding us that we need to pay attention to the impact of anthropogenic disturbances on fish biodiversity and resource. This study can offer scientific references for fisheries management to other river sections of the Jialing River after the fishing ban. Due to the limitations of the study area and data collection, some conclusions drawn from the study still need verification. Therefore, comprehensive and integrated surveys should be conducted in further studies.

## Figures and Tables

**Table 1:** The fish species composition of the study area in the Jialing River sampled from 2021 to 2025. The trophic guild was identified by feeding habits. O represents omnivores, H represents herbivores, I represents invertivores, and P represents piscivores.

Species	*n*	*TL* Range (cm)	*BW* Range (g)	*IRI*	ICUN	Trophic Guild
Cypriniformes						
Catostomidae						
*Myxocyprinus asiaticus*	2	25.8–33.9	204.5–553.7	0.1	CR	I
Cyprinidae						
*Ctenopharyngodon idella*	80	27.6–77.7	295.0–5450.0	124.8	LC	H
*Squaliobarbus curriculus*	2	49.5–53.0	1194.7–1280.3	0.2	LC	O
*Pseudolaubuca sinensis*	6	19.5–25.5	43.5–137.8	0.5	LC	O
*Pseudolaubuca engraulis*	12	15.7–25.2	24.0–96.1	0.9	LC	O
*Hemiculter leucisculus*	49	14.0–26.0	31.9–157.3	15.0	LC	O
*Hemiculter tchangi*	20	21.0–27.5	62.2–173.2	3.9	LC	O
*Chanodichthys oxycephaloides*	1841	19.9–53.0	24.9–1291.8	4044.0	LC	I
*Chanodichthys mongolicus*	42	25.0–57.0	106.6–1475.2	32.2	LC	P
*Culter alburnus*	2	25.8–36.0	131.9–347.5	0.1	LC	P
*Chanodichthys erythropterus*	83	21.6–77.0	97.5–3720.0	128.5	LC	P
*Xenocypris argentea*	3	19.2–22.5	79.1–95.0	0.1	LC	H
*Xenocypris davidi*	571	25.8–58.5	100.0–2360.0	1827.2	LC	H
*Xenocypris fangi*	234	27.3–62.4	186.9–2575.0	350.6	VU	H
*Plagiognathops microlepis*	240	20.2–66.7	71.7–3145.0	719.2	LC	H
*Distoechodon tumirostris*	1	65.9–65.9	2615.2–2615.2	0.1	LC	H
*Megalobrama amblycephala*	1	51.7–51.7	1625.0–1625.0	0.1	LC	H
*Pseudobrama simoni*	382	15.5–37.6	35.2–1499.7	230.2	LC	H
*Hypophthalmichthys molitrix*	15	27.0–57.0	192.7–3500.0	8.4	LC	H
*Hypophthalmichthys nobilis*	48	30.4–110.0	205.6–12015.0	102.5	LC	I
*Hemibarbus labeo*	1245	14.4–38.7	27.7–1442.3	2076.0	LC	O
*Hemibarbus maculatus*	75	16.0–43.2	42.5–845.3	59.5	LC	I
*Sarcocheilichthys sinensis*	4	16.2–18.2	45.4–89.2	0.2	LC	O
*Squalidus argentatus*	8	12.0–16.7	18.3–50.7	0.7	LC	O
*Rhinogobio typus*	86	19.7–37.9	45.6–377.8	37.6	LC	I
*Rhinogobio cylindricus*	3	20.5–24.8	60.6–97.0	0.1	LC	I
*Saurogobio dabryi*	21	12.4–24.5	12.0–91.3	4.7	LC	O
*Saurogobio punctatus*	24	15.6–22.5	21.6–108.3	5.0	---	O
*Spinibarbus sinensis*	27	22.2–60.4	146.3–3040.0	32.1	LC	O
*Onychostoma simum*	32	19.2–57.6	122.7–2320.0	18.6	NT	H
*Onychostoma macrolepis*	1	24.5–24.5	140.2–140.2	0.0	VU	H
*Bangana rendahli*	5	37.6–40.0	612.6–796.6	0.2	DD	H
*Procypris rabaudi*	104	12.6–41.2	24.2–942.7	78.9	VU	O
*Cyprinus carpio*	137	18.5–88.3	92.4–9000.0	656.5	LC	O
*Cyprinus carpio Songpu*	8	20.9–40.2	184.2–950.5	1.7	---	O
*Carassius auratus*	475	15.2–34.6	66.8–705.3	718.8	LC	O
Siluriformes						
Bagridae						
*Tachysurus fulvidraco*	24	14.3–32.1	34.6–205.4	3.5	LC	O
*Tachysurus vachellii*	66	14.9–38.0	20.9–318.6	40.7	LC	O
*Tachysurus nitidus*	9	14.5–24.6	17.4–153.0	0.7	LC	O
*Tachysurus dumerili*	1	25.2–25.2	104.7–104.7	0.0	NT	P
*Tachysurus crassilabris*	209	12.6–33.5	17.4–338.1	146.7	LC	O
*Tachysurus emarginatus*	89	13.5–28.2	23.7–193.0	19.9	DD	I
*Tachysurus pratti*	3	21.1–23.0	76.4–89.1	0.1	VU	I
*Hemibagrus macropterus*	17	17.5–42.3	43.2–414.5	5.2	LC	I
Siluridae						
*Silurus asotus*	21	13.3–66.0	15.6–414.5	11.6	LC	P
*Silurus meridionalis*	6	38.6–54.5	357.5–991.6	1.4	LC	P
Perciformes						
Sinipercidae						
*Siniperca chuatsi*	1078	13.1–31.2	26.0–505.1	1203.3	LC	P
*Siniperca kneri*	47	16.7–28.8	76.2–391.9	10.2	LC	P
*Siniperca scherzeri*	45	15.7–30.9	55.8–390.0	19.2	LC	P
Gobiidae						
*Rhinogobius giurinus*	8	0.9–5.4	2.1–6.2	0.2	LC	I

## Data Availability

Data are contained within the article and will be available upon request.

## Supplementary Materials

The following supporting information can be downloaded at: https://www.mdpi.com/article/10.3390/biology14091208/s1, Figure S1: The phylogeny based on cytochrome *c* oxidase I (COI) for fish species in the study area; Table S1: The list of fish species for phylogeny construction in this study.
