# Supplementary material for: Temporal Variation and Assembly Process of Fish Communities in a Typical Canalized River After the 10-Year Fishing Ban
Source: Biology (Basel). 2025 Sep 7;14(9):1208. doi: 10.3390/biology14091208 (PMC12467309; doi:10.3390/biology14091208)
Supplement: Supplementary file 1 [file biology-14-01208-s001.zip › biology-3802372-supplementary.pdf]

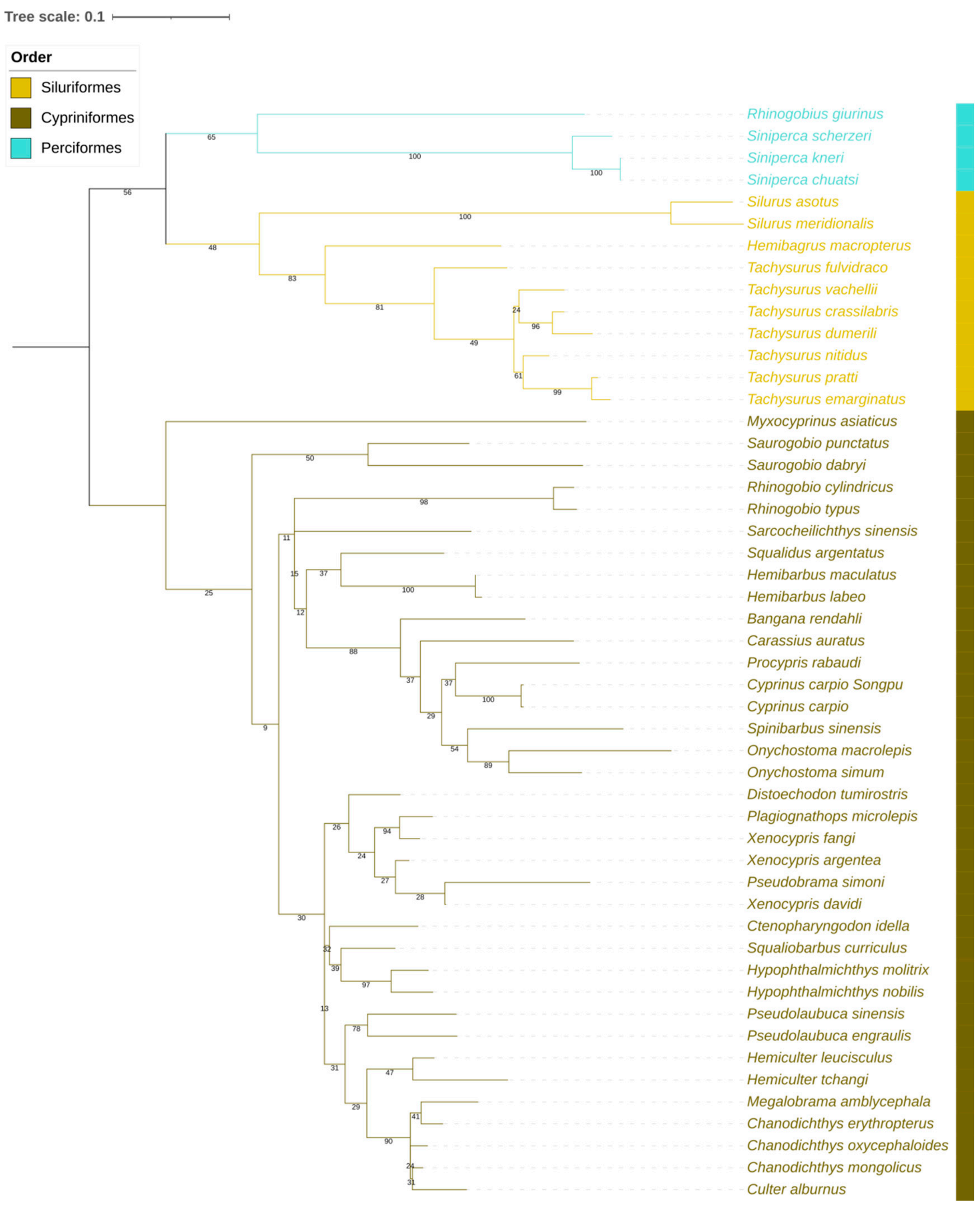

Figure S1. The phylogeny based on cytochrome *c* oxidase I (COI) for fish species in the study area.

Table S1. The list of fish species for phylogeny construction in this study.

Order	Species	Accession number	Size（bp）
Cypriniformes	*Myxocyprinus asiaticus*	MF122546.1	624
Cypriniformes	*Ctenopharyngodon idella*	MT804981.1	561
Cypriniformes	*Squaliobarbus curriculus*	OP051035.1	647
Cypriniformes	*Pseudolaubuca sinensis*	MT805704.1	561
Cypriniformes	*Pseudolaubuca engraulis*	OP050729.1	647
Cypriniformes	*Hemiculter leucisculus*	OP050543.1	647
Cypriniformes	*Hemiculter tchangi*	MZ870937.1	585

Order	Species	Accession number	Size（bp）
Cypriniformes	*Chanodichthys oxycephaloides*	MF122236.1	624
Cypriniformes	*Chanodichthys mongolicus*	KR861856.1	624
Cypriniformes	*Culter alburnus*	KR861869.1	624
Cypriniformes	*Chanodichthys erythropterus*	MZ403252.1	602
Cypriniformes	*Xenocypris argentea*	KR862281.1	624
Cypriniformes	*Xenocypris davidi*	MZ871361.1	585
Cypriniformes	*Xenocypris fangi*	MF123401.1	624
Cypriniformes	*Plagiognathops microlepis*	MF805675.1	623
Cypriniformes	*Distoechodon tumirostris*	GU434342.1	652
Cypriniformes	*Megalobrama amblycephala*	HM776368.1	677
Cypriniformes	*Pseudobrama simoni*	MZ870981.1	585
Cypriniformes	*Hypophthalmichthys molitrix*	OP050549.1	647
Cypriniformes	*Hypophthalmichthys nobilis*	MT805177.1	561
Cypriniformes	*Hemibarbus labeo*	MN267847.1	577
Cypriniformes	*Hemibarbus maculatus*	MT571843.1	672
Cypriniformes	*Sarcocheilichthys sinensis*	KR862170.1	624
Cypriniformes	*Squalidus argentatus*	OP050867.1	647
Cypriniformes	*Rhinogobio typus*	MZ871000.1	585
Cypriniformes	*Rhinogobio cylindricus*	MZ870993.1	585
Cypriniformes	*Saurogobio dabryi*	MZ871101.1	585
Cypriniformes	*Saurogobio punctatus*	ON041156.1	642
Cypriniformes	*Spinibarbus sinensis*	MZ871179.1	585
Cypriniformes	*Onychostoma simum*	MH193224.1	598
Cypriniformes	*Onychostoma macrolepis*	MH193207.1	598
Cypriniformes	*Bangana rendahli*	MF122084.1	624
Cypriniformes	*Procypris rabaudi*	MF122640.1	624
Cypriniformes	*Cyprinus carpio*	MT805027.1	561
Cypriniformes	*Cyprinus carpio Songpu*	KC108650.1	628
Cypriniformes	*Carassius auratus*	MT804949.1	561
Siluriformes	*Tachysurus fulvidraco*	MT571911.1	672
Siluriformes	*Tachysurus vachellii*	MN639822.1	620
Siluriformes	*Tachysurus nitidus*	MH317165.1	674
Siluriformes	*Tachysurus dumerili*	MZ870942.1	585
Siluriformes	*Tachysurus crassilabris*	MH317170.1	674
Siluriformes	*Tachysurus emarginatus*	MN660222.1	533
Siluriformes	*Tachysurus pratti*	MN639838.1	620
Siluriformes	*Hemibagrus macropterus*	MZ870742.1	585
Siluriformes	*Silurus asotus*	MZ871103.1	585
Siluriformes	*Silurus meridionalis*	MZ871114.1	585
Perciformes	*Siniperca chuatsi*	MF123224.1	624
Perciformes	*Siniperca kneri*	EF143389.1	666
Perciformes	*Siniperca scherzeri*	OP050852.1	647
Perciformes	*Rhinogobius giurinus*	KM610889.1	648
